# Knowledge of congenital cytomegalovirus (cCMV) among physical and occupational therapists in the United States

**DOI:** 10.1371/journal.pone.0185635

**Published:** 2017-10-04

**Authors:** Kathleen M. Muldoon, Amy Armstrong-Heimsoth, Jodi Thomas

**Affiliations:** 1 Department of Anatomy, Arizona College of Osteopathic Medicine, Midwestern University, Glendale, Arizona, United States of America; 2 Department of Occupational Therapy, Northern Arizona University, Phoenix, Arizona, United States of America; 3 Department of Physical Therapy, Midwestern University, Glendale, Arizona, United States of America; University of St Andrews, UNITED KINGDOM

## Abstract

Congenital cytomegalovirus (cCMV) infections cause more children to have permanent disabilities than Down Syndrome, Fetal Alcohol Syndrome, Spina Bifida, and pediatric HIV/AIDS combined. The risk of infection during pregnancy can be significantly decreased using universal precautions, such as thorough handwashing and cleansing of surfaces and objects that have come into contact with infected body fluids. Children under 3 years of age are commonly asymptomatic excretors of CMV, with the highest viral loads present in saliva. Pediatric therapists have regular close contact with young children, and are thus likely at elevated occupational risk of acquiring CMV. Our objective was to evaluate therapist knowledge of cCMV and its transmission. We recruited American Occupational Therapy Association (AOTA) and American Physical Therapy Association (APTA) members via electronic newsletters and printed flyers from April to September 2015. Participants completed an online, anonymous 24-question survey using Survey Monkey. We compared responses between groups and previously published CMV awareness data using binomial tests of difference of proportions and multiple logistic regression. Our study identified both a low level of therapist awareness and poor demonstrated understanding of cCMV. Self-reported cCMV awareness amongst therapists was greater than awareness in the general population, and equivalent to awareness amongst health care professionals. Whereas 52% of participants self-reported awareness of cCMV, only 18% demonstrated understanding of the behavioral modes of CMV transmission. Fewer therapists reported awareness of cCMV than other, less prevalent conditions. Higher levels of health risk knowledge were associated with greater contact with children. Most participants reported learning about cCMV from the workplace. The knowledge gaps between self-reported awareness of cCMV and demonstrated understanding of modes of transmission described by our results emphasize the need for additional training of therapists. cCMV is preventable, and accurate knowledge of modes of transmission is crucial for the health of practitioners and clients.

## Introduction

Cytomegalovirus (CMV) is a common virus that remains latent in multiple organs, and is periodically excreted throughout an individual’s life [1]. CMV is transmitted from person to person through close contact with CMV-infected body fluids; predominantly saliva, urine, tears, and genital secretions. CMV can also be transmitted from mother to child across the placenta or through breastmilk [2, 3]. CMV infection in healthy adults and children is usually mild or asymptomatic. However, maternal transmission to her fetus or newborn can cause significant visceral damage [4]. cCMV is a leading cause of prenatal infection worldwide, and the most common congenital infection in the United States [5, 6]. In the US, approximately 40,000 newborns are diagnosed with cCMV annually, resulting in an estimated 400 deaths, and 8,000–10,000 children with permanent disabilities, including cognitive impairments, movement disorders, and hearing and/or vision loss [4, 7]. More children have disabilities due to cCMV than other well-known infections and syndromes, including Down Syndrome, Fetal Alcohol Syndrome, Spina Bifida, and Pediatric HIV/AIDS combined [8].

Approximately 10–15% of infected newborns show CMV-specific clinical symptoms at birth, such as hearing loss, hepatosplenomegaly, jaundice, petechiae, and/or microcephaly [9, 10]. Of the infected newborns who are asymptomatic, up to 14% develop permanent disabilities later in life [11], such as deafness, cerebral palsy, developmental delays, and seizures. These data are likely underestimates of the true prevalence of cCMV because newborns are not universally screened for the virus [12, 13], data are lacking on important outcomes, such as visual impairment and hearing loss [14–16], and follow-up of congenitally infected children has been too short to identify late-onset outcomes [17].

Infected individuals, especially children less than 3 years of age, may excrete (shed) CMV in saliva and urine for months to years after initial infection [18, 19]. Viral loads of CMV are higher in the saliva than the urine of shedding children [20], indicating that exposure to saliva poses the greatest risk for acquiring CMV. Furthermore, viable CMV can persist on environmental surfaces, such as toys, food, and countertops, for up to 6 hours, which is long enough to pose a transmission risk [21]. CMV infection rates are estimated to be up to 80% in otherwise healthy toddlers, who are at high risk for contracting CMV from their peers in high-risk locations, such as homes, daycares, schools, and pediatric health care centers [22].

Contact with the body fluids of young children is a major cause of CMV infection among women of reproductive age. CMV infection during pregnancy can occur as a result of either a primary (first-time) or recurrent infection (reactivation of a latent virus, or reinfection from a new strain of CMV) [23, 24]. The risk of becoming infected depends primarily on two factors: the frequency of contact with a vector of CMV and the likelihood that any given interaction will be with a person who is shedding CMV in their bodily fluids [25]. Therefore, it is not surprising that professional activity correlates with the probability of acquiring CMV, particularly in careers that involve working with young children who are commonly asymptomatic excretors of the virus. Although the occupational risk of CMV contamination was contested historically [26, 27], elevated rates of CMV acquisition in daycare providers, teachers, nurses’ aides, and other pediatric healthcare workers has been repeatedly demonstrated [28–37].

Because there is currently no CMV vaccine [38], it is imperative that women of childbearing age who have close contact with young children be informed of behaviors that help to prevent cCMV infection. Exposure to CMV can be reduced through standard universal precautions: thorough handwashing following contact with children or handling objects such as children’s toys, and thorough cleansing of surfaces that have come into contact with children’s bodily fluids [39]. Research on the impact of educational interventions support the positive effect of preventative strategies [40, 41]. A recent study found that only 1.2% of pregnant women who were given information about CMV acquired the infection before delivery, in contrast to 7.6% that were not informed about CMV while pregnant [42].

Even though CMV infection in mothers is preventable, most people are not familiar with the virus. Levels of CMV awareness amongst women in the general population range from 9%-20%, globally [7, 42–46], as measured by self-reported responses to questionnaires. Some studies have shown a statistically significant increase in women’s awareness with higher levels of education (23–31% in college-educated women [44]), or work experience in health care (56%; [44]). Interestingly, neither having been pregnant nor employed in a daycare setting has a statistical effect on CMV awareness [44]. Overall CMV awareness remains lower than other less prevalent childhood illnesses, even among medical and allied health professionals [47–49].

Moreover, previous work likely overestimates cCMV awareness levels for two main reasons. Firstly, participants often exaggerate responses in self-report studies [50] Social desirability bias describes the tendency for survey respondents to answer questions in a way that would be viewed favorably by others, such as indicating awareness of a condition for which they actually have no knowledge. Secondly, awareness is a subjective form of knowledge, and can mean different things to different people. For example, a research participant may have heard of CMV, or recognize it from a list of chronic health conditions, yet be unable to identify the detrimental effects of a congenital infection on a newborn, or modes of CMV transmission. In other words, participants may be aware of CMV, but their demonstrated understanding of CMV is inadequate to prevent infection while pregnant. Demonstrated understanding is thus a higher-level, objective form of knowledge. Most previous work on cCMV knowledge describes only awareness levels of cCMV. However, Jeon and colleagues (2006) reported that even women who reported being aware of CMV could not identify modes of CMV transmission or clinical outcomes of a congenital infection [44]. It is not enough that women of childbearing age are aware of cCMV; they must also demonstrate understanding of the health risks of cCMV in order for public health interventions to be successful [16, 51].

CMV awareness studies have generally surveyed either the general population [7, 42–45], or facility-specific health-care staff, such as nurses or midwives [26–37]. To date, no published work has examined cCMV knowledge amongst therapists. Physical therapy (PT) and occupational therapy (OT) are forms of rehabilitative health. The goal of PT is to maximize functional control of the body, or increase gross motor function, by training and strengthening a patient’s large muscles (those in the arms, legs, and abdomen). The goal of OT is to develop an individual’s ability to function in their typical daily activities in order to foster independence, productivity, and self-care. In doing so, OTs focus on strength, dexterity and coordination while performing tasks. Both physical and occupational therapy involve physical handling and repeated close contact, particularly in subfields that focus on work with infants and children. Despite small fluctuations in demographic profiles, PTs and OTs are predominantly female (70% of PTs in 2013, and 91% of OTs in 2015), white (92% of PTs, and 85% of OTs), and of childbearing age (51% of PTs are between 20–45 years old, and 53% of OTs are between ages of 30–39) [52] [53]. It is difficult to estimate the number of therapists that identify pediatrics as their primary subfield of practice, as this data has not been systematically gathered by national associations (C. Sliwa, pers. comm). Therapists who work with children do so in many different settings (e.g., hospitals, schools, outpatient rehabilitation centers, early intervention programs), and may not work with children exclusively [51, 52]. However, physical and occupational therapy are essential components of a multidisciplinary approach to treating children with special needs, such as cerebral palsy and other movement disorders [54].

Pediatric PTs and OTs have regular close contact with young children, as well as the toys, supports, and other surfaces that may be subject to their bodily fluids, particularly saliva. Pediatric therapists are thus not only exposed to numerous pathogens throughout their workday, but may also serve as sources of infection to their patients [55]. Given higher rates of CMV acquisition in occupations that focus on the care of young children, pediatric therapists are likely at higher occupational risk of CMV exposure [56], although a study of CMV seroconversion rates within this population has not yet been conducted. Previous evidence suggests that OTs have little knowledge of CMV and its relationship to infection control practices [57]. For example, the literature suggests that sharing of toys by children in daycare may be a reason for the high incidence of CMV in these settings [36]. However, Flacker (1993) found that only 15% of respondents reported always disinfecting equipment after it has been used, and only 23% reported disinfecting toys after contact with patient [57]. Furthermore, although universal precautions are widely accepted as key to infection prevention, Marcil (1993) found that only 66% of OTs washed their hands after patient contact [55]. Pediatric therapists must therefore be considered an at-risk population for CMV infection. Considering the number of children affected each year by cCMV, the fact that young children are both asymptomatic excretors of CMV and shed the virus at higher rates than other groups, and that the virus spreads easily in pediatric facilities, it is important to educate therapists, especially those who work with children, to reduce cCMV infections.

The aims of this study are to threefold. First, we aim to quantify PT and OT knowledge of cCMV. Our study is the first to distinguish cCMV awareness and cCMV demonstrated understanding as discrete forms of cCMV knowledge. Given the results of previous cCMV knowledge surveys, we hypothesize that PTs and OTs will have 1) low overall cCMV knowledge, but 2) more knowledge than the general population, and 3) equal knowledge to health care workers. We further hypothesize that PTs and OTs will have a lower demonstrated understanding of cCMV than their self-reported awareness. Secondly, we aim to describe gaps in the cCMV knowledge amongst PTs and OTs that represent opportunities for education. Thirdly, we aim to define the variables that drive cCMV knowledge within the therapist community. Given that our sample includes only respondents with training in allied health sciences, we hypothesize that cCMV knowledge will vary by age, time in practice, specialty, and amount of contact with children. These data will inform our understanding of variance in cCMV knowledge amongst therapists, augment the role of therapists in preventing infectious disease transmission, and improve therapeutic practice for practitioners at high-risk for CMV acquisition.

## Methods

### Participants

We targeted American Physical Therapy Association (APTA), and American Occupational Therapy Association (AOTA) members to participate in our study. We recruited participants by publishing a brief description of the study and link to the online survey to the following venues: 1) the pediatric section of the APTA monthly electronic newsletter; 2) the neurology section of the APTA electronic listserv; 3) the AOTA newsletter *OT Practice*; 4) the AOTA managed forum *OT Connections*; and 5) by printed flyer to attendees of the national 2015 annual conference of the AOTA in Nashville, TN. We accepted responses from any licensed PT, PT assistant (PTA), PT student (PTS) or OT, OT assistant (COTA), or OT student (OTS) who volunteered to complete the survey. We recruited participants across physical and occupational therapy, including those who work with children, as knowledge of cCMV is particularly important in these populations. We did not offer compensation for participation. Participation was anonymous, voluntary, and free of coercion. We obtained informed consent on page one of the online survey (S1 Appendix, Question 1). Subjects created their own unique identifier to maintain confidentiality (S1 Appendix, Question 2). Our study was approved by the Institutional Review Board of Midwestern University (MWU Project AZ #823, IRB approval 03/03/2015).

### Survey design and instrumentation

We designed a 24 question, 10-minute survey to assess participant demographics, work experience, general awareness and perceived occupational hazard of several chronic and acute health conditions, specific knowledge of cCMV (including transmission, clinical outcomes, and treatment), and the source of participant knowledge of cCMV (see S1 Appendix for the complete questionnaire and summary data for results presented in this paper). We modeled the design of the survey on previously published questionnaires assessing CMV awareness amongst medical students [47]. We solicited information about several demographic parameters. We asked participants their occupation to determine their professional role within physical or occupational therapy (S1 Appendix, Question 3). We inquired if participants work with children, and the percentage of their caseload that is with children (for example, a pediatric PT may work with children for 100% of their caseload, but a neurologic PT may also carry a partial pediatric caseload; S1 Appendix; Questions 4–5). We also asked their years of experience, and years of experience working with children (S1 Appendix, Questions 6–7). We collected these data to address whether the degree to which participants work with children, and/or amount of professional experience had an effect on therapist knowledge of cCMV. We asked participants if they are parents, and, if so, of how many children, respectively (S1 Appendix, Questions 17–18). We used these data to examine whether being a parent had an effect on cCMV knowledge, beyond the professional subfield of the participant. We asked participants the frequency of their contact with children five years of age or younger (S1 Appendix, Question 19), in order to assess whether contact with children had an effect cCMV knowledge independent of being a parent. We also asked participants to identify the source of their cCMV knowledge (S1 Appendix, Question 20), in addition to basic demographic questions (age, gender, race/ethnicity, and domestic status) in questions 21–24 (S1 Appendix).

**Fig 1:**
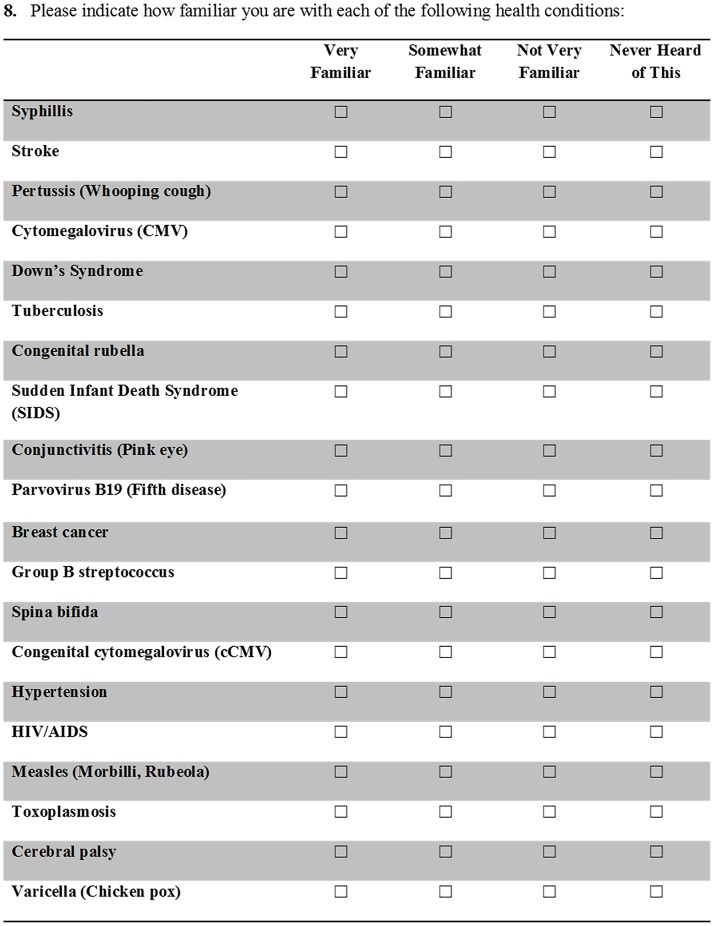
We asked participants to rank their familiarity with health conditions on a 4-point scale (S1 Appendix, Question 8) in order to quantify cCMV awareness levels. We recoded these data to calculate the summary variable Self-Reported Familiarity (SRF).

**Fig 2:**
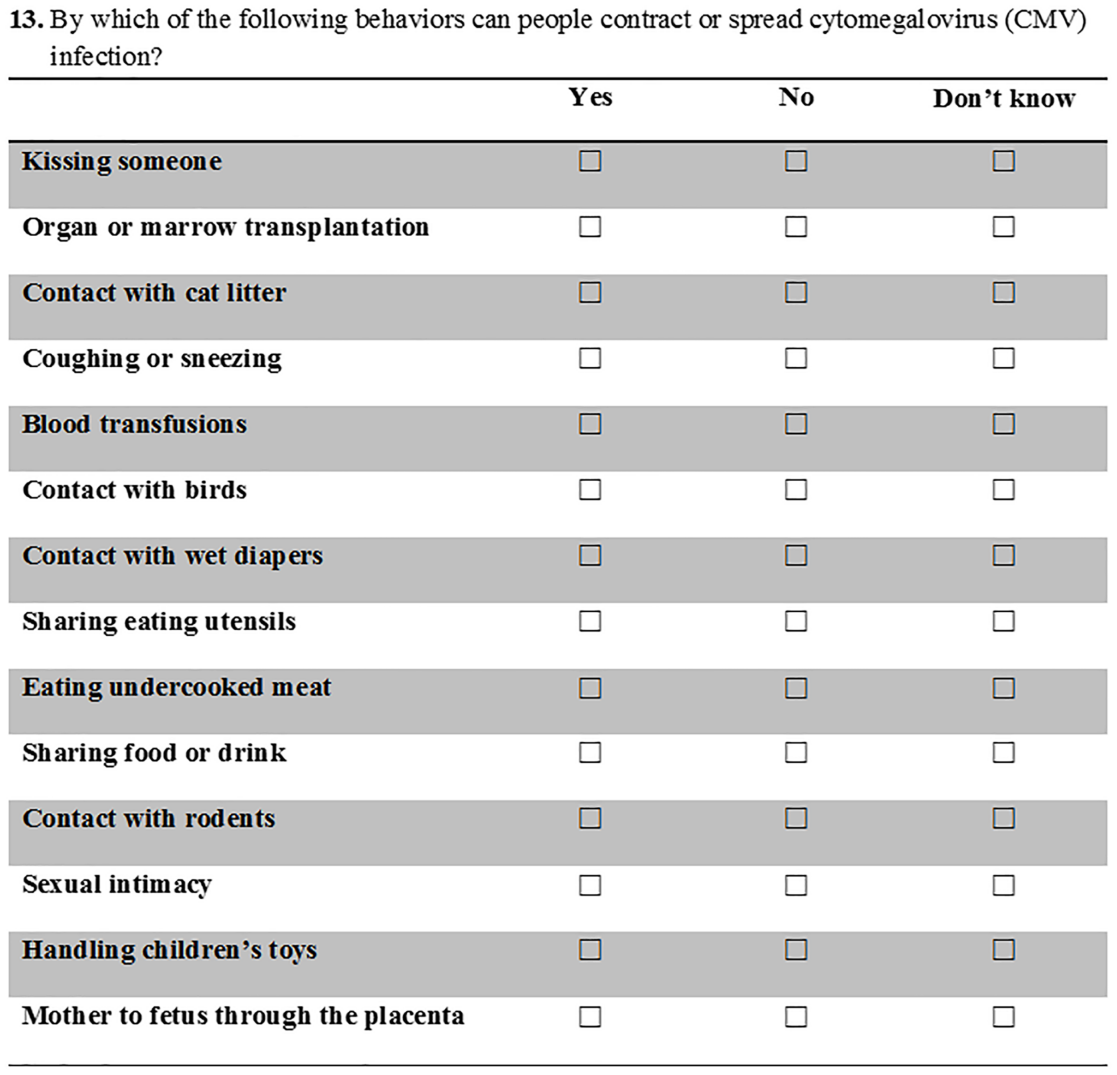
We asked participants to describe their understanding of CMV transmission (S1 Appendix, Question 13). We used these data to examine the demonstrated understanding of participant Health Risk Knowledge (HRK) regarding cCMV.

We constructed questions 8–16 to quantify levels of knowledge about CMV. For questions of awareness of health conditions, we asked participants to rank their familiarity or concern with common conditions and illnesses on a 4-point scale (Very Familiar, Somewhat Familiar, Not Very Familiar, or Never Heard of This; e.g., S1 Appendix, Question 8, Fig 1). To assess demonstrated understanding of specific knowledge and behaviors, we asked participants to answer either Yes, No, or Don’t Know (e.g., S1 Appendix, Question 13, Fig 2). Proposed response options for each question were based on the literature [47, 58] and some were false (e.g., symptoms and modes of transmission not associated with CMV).

Our online questionnaire was administered through Survey Monkey [59], and remained open for six months (April-September 2015). We chose an online platform to administer the survey to maximize sample size and minimize cost. However, the lack of control over an online platform has led to commonly observed problems that may affect data quality [60, 61]. Most research exploring the suitability of the internet for conducting surveys focusses on the use of online labor markets (e.g. [60, 62, 63]). In contrast to surveys that recruit convenience samples through crowdsourcing, we used traditional targeted sampling methods to recruit professional therapists and therapy students, only. We did not recruit participants through Survey Monkey. Furthermore, we adopted the best research practices developed by Chandler and Shapiro (2016) when designing our survey to minimize the limitations associated with online research [60]. Firstly, studies have demonstrated that survey participants will act deceptively if incentivized to do so, leading to errors resulting from the non-naiveté and trustworthiness of participants [60, 64]. We did not offer compensation for participation, since data quality is generally unaffected by pay when participants are asked to self-report answers rather than to complete a task [65, 66]. Secondly, we did not inform prospective participants that cCMV was the primary topic of the study, but that the survey topic was about health risks in therapeutic practice. In this way, we converted any potential unmeasurable selection bias caused by the topic of study into measurable study attrition [60]. Thirdly, we reduced and measured study attrition. We minimized the effect of selective attrition on our results by placing the most critical survey questions early in the survey, so that those who dropped out could be excluded even if they did not complete the study [67]. We also assessed the robustness of our study to attrition by comparing the demographic profiles of respondents at the beginning of our survey with the demographic profiles of respondents to each key research question (S2 Appendix). Fourthly, we minimized repeated participation by using Survey Monkey platform settings to restrict multiple responses. Participants also created their own unique identifiers, and Survey Monkey tracked the IP addresses of respondents.

### Knowledge metrics

In addition to assessing responses on the 4-point scale presented in our survey, we created two summary variables to capture variation in therapist knowledge of cCMV. We created Self-Reported Familiarity (SRF) to compare our data with previously published research on cCMV awareness, which take the binary form of “aware/unaware” [44], or “% who have heard of cCMV” [42, 46]. We considered participants who answered “Very Familiar” or “Somewhat Familiar” to the question, “Please indicate how familiar you are with each of the following health conditions” to have high SRF, and those who answered “Not Very Familiar” or “Never Heard of it” to have low SRF (S1 Appendix, Question 8, Fig 1).

To describe PT and OT cCMV knowledge in relation to published work, we relied predominantly on data from two previously published surveys of cCMV awareness in the United States. Jeon et al. (2006) surveyed 643 women at seven geographic locations, representing a diverse population of women including medical students and hospital support staff. Participants were asked to “Identify which of the following diseases you have heard of” from a list that included congenital CMV, Parvovirus B19, congenital Toxoplasmosis, congenital Rubella Syndrome, Group Beta Streptococcus (GBS), Spina Bifida, Fetal Alcohol Syndrome (FAS), Sudden Infant Death Syndrome (SIDS), Down Syndrome, and HIV/AIDS [44]. The authors grouped responses by level of education, as well as previous employment as a healthcare professional. They found that 31% of women with a bachelor’s degree, 24% of women who had completed graduate school, and 56% of women with work experience in health care had heard of CMV. A limitation of this work is that Jeon et al (2006) administered the survey mainly in healthcare settings.

Doutré et al (2016) updated results presented in Ross et al (2008), by analyzing results of the 2015 and 2016 HealthStyles survey [42, 45]. The HealthStyles survey is a consumer mail survey of US adults over 18 years of age, commonly used by the Centers for Disease Control and Prevention (CDC) for public health planning. The survey asked, “Have you heard of the following?” Responses included: Congenital Rubella Syndrome (CRS), Group Beta Streptococcus (GBS), HIV/AIDS, cCMV, Down Syndrome, Sudden Infant Death Syndrome (SIDS), Fetal Alcohol Syndrome (FAS), Autism, Spina Bifida, congenital toxoplasmosis, and Parvovirus B19 (Fifth Disease) [45]. The data were weighted to create a nationally representative sample with respect to demographic characteristics, including level of educational attainment. They found that in a mixed sex sample, 7% of the overall population, 10% of participants with a bachelor’s degree, and 20% of those with a professional degree or doctorate were aware of CMV. Awareness among women was 9% [45], which was statistically lower than previously reported values from the 2005 HealthStyles survey [42, 45], although this decrease may be due to sampling error. We consider the data derived from Jeon et al. (2008) and Doutré et al. (2016) to be comparable to the data derived from Question 8 of our survey, (e.g., “Please indicate how familiar you are with each of the following health conditions”, S1 Appendix).

In addition, we calculated a new variable called Health Risk Knowledge (HRK) to represent participants who demonstrated understanding of cCMV by correctly answered the question, “By which of the following behaviors can people contract of spread cytomegalovirus (CMV) infection?” (S1 Appendix, Question 13, Fig 2). We counted participants as having a high degree of HRK if they correctly identified all behavioral modes of CMV transmission related to close contact (e.g., kissing someone, contact with wet diapers, sharing eating utensils, sharing food or drink, handling children’s toys), even if they did not correctly identify medical modes of transmission of CMV (e.g., organ or marrow transplant, blood transfusions, mother to fetus across the placenta). This is because close contact with infected bodily fluids poses the greatest risk of CMV transmission to therapists. We considered those who could not identify all behavioral modes of CMV transmission to have low HRK. HRK removes the effect of response bias [50], whereby participants may have over-reported their familiarity. Although we preferred HRK as a stronger, objective form of knowledge, and as a metric for demonstrated understanding of cCMV, SRF is comparable with prior research on cCMV awareness. Therefore, we present both measures of knowledge in relation to our hypotheses and descriptive statistics.

### Data analysis

To test the hypothesis that PTs and OTs have more knowledge of cCMV than the general population, we compared the percentage of our survey respondents with high SRF (e.g., awareness) and high HRK (e.g., demonstrated understanding) with previously published cCMV awareness levels amongst women in the general US population (9%, [45]). We also compared our data to awareness levels in women who had graduated college (31%, [44]) and graduate school (23%, [42]) We made these comparisons to evaluate CMV knowledge across individuals of similar levels of educational attainment, but different fields of study. Because therapists are allied health professionals, we tested the hypothesis that PTs and OTs have equivalent knowledge of cCMV to healthcare workers by comparing our data with published percentages of cCMV awareness levels amongst women with experience working in health care [44]. We omitted male respondents from all comparisons (N = 9, 4.5% of our total sample) because most previous work reports only CMV awareness amongst women. For all comparisons, we used binomial tests of difference of proportions. We ran analyses using all participants in our survey, as well as for PTs and OTs, separately. However, we consider the sample size for OTs too low to yield reliable results.

We used descriptive statistics to examine the relationship between SRF and HRK and participant understanding of the clinical manifestations of cCMV. We used multiple logistic regression to examine which variables were the most important drivers of variation in HRK in the entire sample, and in PTs and OTs separately. We selected *a priori* only predictor variables with significant univariate associations with HRK to enter into the logistic regression. We ran all analyses using SPSS 22.0 for Windows [68].

## Results

Our total sample size was 230 respondents. Question-specific sample sizes ranged from 182 to 215 (S1 Appendix). The majority of participants were PTs, PTAs, or PT students (N = 176, 82%), white (N = 168, 92.8%), female (N = 170, 93.4%), and parents (N = 112, 61.5%). Overall, the mean age range of respondents was 35–44 years old. The mean years’ work experience was 20.1 years (range = 0–44 years), and mean years’ work experience with children was 13.5 years (range = 0–44 years). Most respondents worked with children for some part of their caseload (N = 165, 76.7%), with 60.5% (N = 130) of respondents indicating that greater than 75% of their caseload was working with children. Overall, the demographic distribution of respondents adequately reflects the membership profiles of practicing therapists, as reported by APTA [52] and AOTA [53], although our sample is skewed towards pediatric therapists. We considered our sample to be a conservative estimate of cCMV awareness in the broader PT and OT community, because it may expected that therapists working with children have greater familiarity with congenital infections than therapists working with adults. Not all respondents completed all questions of the survey. However, the demographic profile of respondents to the initial survey question was statistically indistinguishable from those of each subsequent survey question. As such, survey attrition did not bias our results (S2 Appendix).

Prior to data analysis, we examined responses for duplicate identifiers or IP addresses. We found one instance in which the same IP address was associated with eight different responses (~3.5% of our sample), each with a unique identifier. This is slightly higher than the published average rate of 2.5–2.8% of respondents who share an IP address with at least one other worker in online studies [69, 70]. However, closer examination revealed that these eight workers reported occupations, caseloads, years of experience, and demographic characteristics that were consistent with being distinct professionals in a single place of work. It is likely that these responses represent multiple individuals completing the survey using a single work computer.

**Fig 3:**
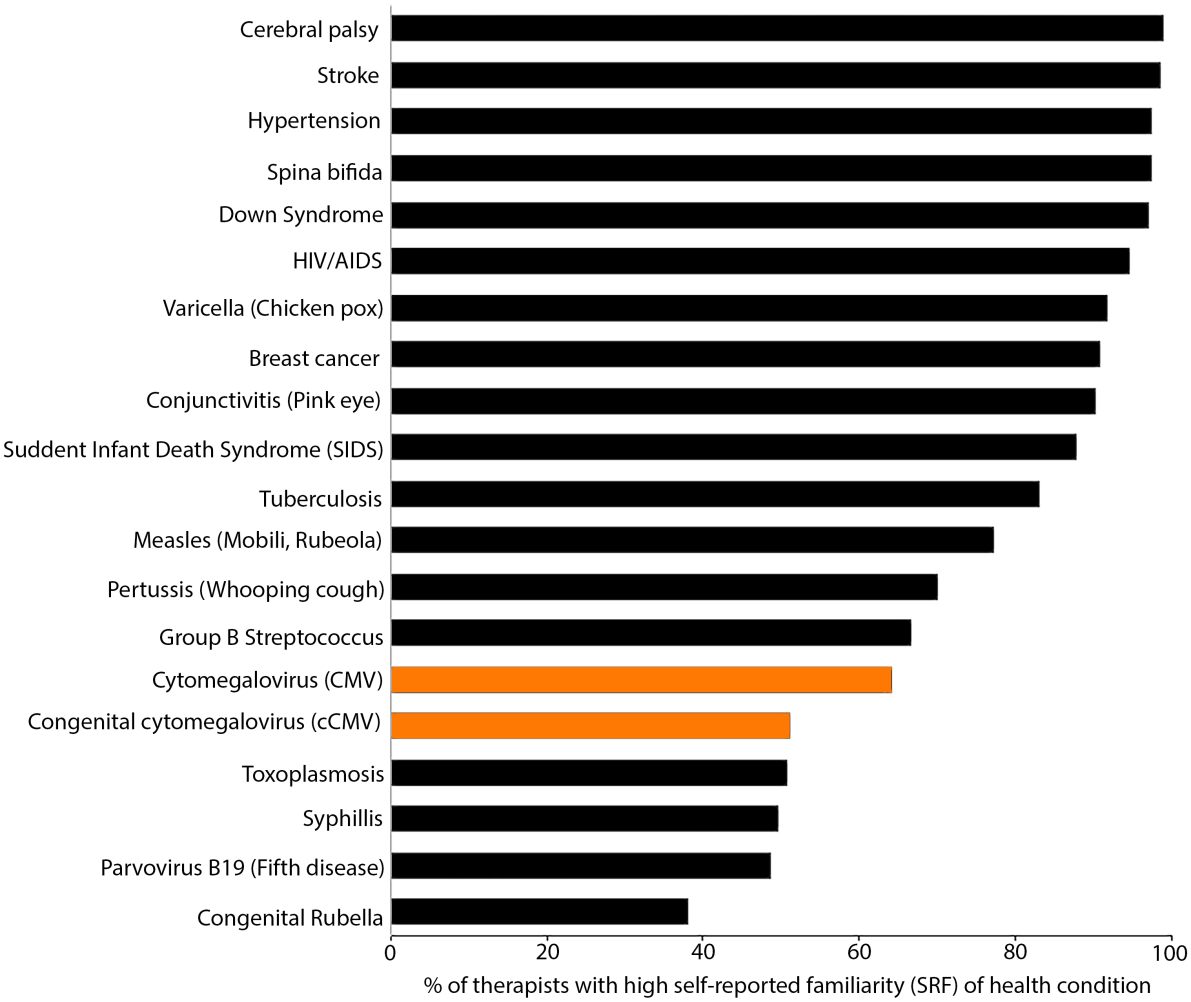
Awareness (measured as Self- Reported Familiarity [SRF]) of chronic and acute health conditions amongst physical and occupational therapists.

Using the full 4-point scale of responses for Question 8, the median rank for familiarity with health conditions was “Somewhat Familiar” across the entire sample (Table 1). Responses were skewed to the left (unfamiliar) end of the scale, although respondents used the full range of possible responses for only 7/20 conditions (Group B Streptococcus, CMV, cCMV, Parvovirus B19 [Fifth Disease], Toxoplasmosis, congenital Rubella, and Varicella [Chicken Pox]). These are the only conditions for which some respondents chose “Never Heard of This”. Of 201 female respondents, 105 (52%) were aware (had high SRF) of cCMV (Table 2). Relative to other health conditions included in the survey, SRF of CMV and cCMV ranked 15^th^ and 16^th^ out of 20 (Fig 3).

**Table 1:** Respondent familiarity with health conditions and illnesses (N = 207). Proportion of respondents given in parentheses. * indicates most common response.

HEALTH CONDITION	VERY FAMILIAR	SOMEWHAT FAMILIAR	NOT VERY FAMILIAR	NEVER HEARD OF THIS
**Syphillis**	10 (4.8)	93 (44.9)	104 (50.2)*	0
**Stroke**	177 (85.5)*	27 (13.0)	3 (1.4)	0
**Pertussis (Whooping Cough)**	36 (17.4)	109 (52.6)*	62 (30.0)	0
**Cytomegalovirus (CMV)**	45 (21.7)	88 (42.5)*	56 (27.1)	18 (8.7)
**Down's Syndrome**	156 (75.4)*	45 (21.7)	6 (2.9)	0
**Tuberculosis**	48 (23.2)	124 (59.9)*	35 (16.9)	0
**Congenital Rubella**	16 (7.7)	63 (30.4)	113 (54.6)*	15 (7.2)
**Sudden Infant Death Syndrome (SIDS)**	90 (43.5)	92 (44.4)*	25 (12.1)	0
**Conjunctivitis (Pink Eye)**	116 (56.0)*	71 (34.3)	20 (9.7)	0
**Parvovirus B19 (Fifth Disease)**	36 (17.4)	65 (16.9)	71 (34.3)*	35 (16.9)
**Breast Cancer**	103 (48.8)*	85 (41.1)	19 (9.2)	0
**Group B Streptococcus**	46 (22.2)	92 (44.4)*	62 (30.0)	7 (3.4)
**Spina bifida**	138 (66.7)*	64 (30.9)	5 (2.4)	0
**Congenital Cytomegalovirus (cCMV)**	43 (20.8)	63 (30.4)	70 (33.8)*	31 (15.0)
**Hypertension**	164 (79.2)*	38 (18.4)	5 (2.4)	0
**HIV/AIDS**	109 (52.7)*	87 (42.0)	11 (5.3)	0
**Measles (Morbilli, Rubeola)**	63 (30.4)	97 (46.9)*	47 (22.7)	0
**Toxoplasmosis**	21 (10.1)	84 (40.6)*	81 (39.1)	21 (10.1)
**Cerebral Palsy**	172 (83.1)*	33 (15.9)	2 (1.0)	0
**Varicella (Chicken Pox)**	121 (58.5)*	69 (33.3)	16 (7.7)	1 (0.5)

**Table 2:** Binomial tests of differences of proportion for therapists with high Self-Reported Familiarity (SRF) and high Health Risk Knowledge (HRK) of congenital cytomegalovirus (cCMV) with previously published data on cCMV awareness*.

		Obs. %	General Population (9%)^a^	College (31%)^b^	Graduate School (23%)^a^	Health Care workers (56%)^b^
			*P*-value	*P*-value	*P*-value	*P*-value
SRF	All Sample (N = 201)	52.2 (N = 105)	< 0.001	< 0.001	< 0.001	0.094
	PTs (N = 160)	56.2 (N = 90)	< 0.001	< 0.001	< 0.001	0.477
	OTs (N = 26)	38.5 (N = 10)	0.002	0.265	0.049	0.055
HRK	All Sample (N = 201)	17.7 (N = 32)	< 0.001	< 0.001	0.050	< 0.001
	PTs (N = 164)	18.8 (N = 27)	< 0.001	0.001	0.132	< 0.001
	OTs (N = 26)	11.1 (N = 3)	< 0.001	< 0.001	< 0.001	0.001

*We omitted male respondents since most previous work reports levels of CMV awareness in women, only

^a^ [45]

^b^ [44]

A significantly greater percentage of therapists had high SRF than awareness levels amongst women in the general US population, women who had graduated college, and women who completed 5–8 years of graduate school (Table 2). The percentage of therapists with high SRF in our study could not be distinguished from previously reported levels of CMV awareness in women with experience as health care workers. When analyzed separately, 56% of PTs had high SRF. This proportion was also significantly greater in comparisons across all groups, but could not be distinguished from cCMV awareness in women with experience as health care workers (Table 2). The percentage of OTs with high SRF (39%) was significantly higher than women in the general US population, and women who had completed 5–8 years of graduate school, but indistinguishable from women who had graduated college, and health-care workers (Table 2).

**Fig 4:**
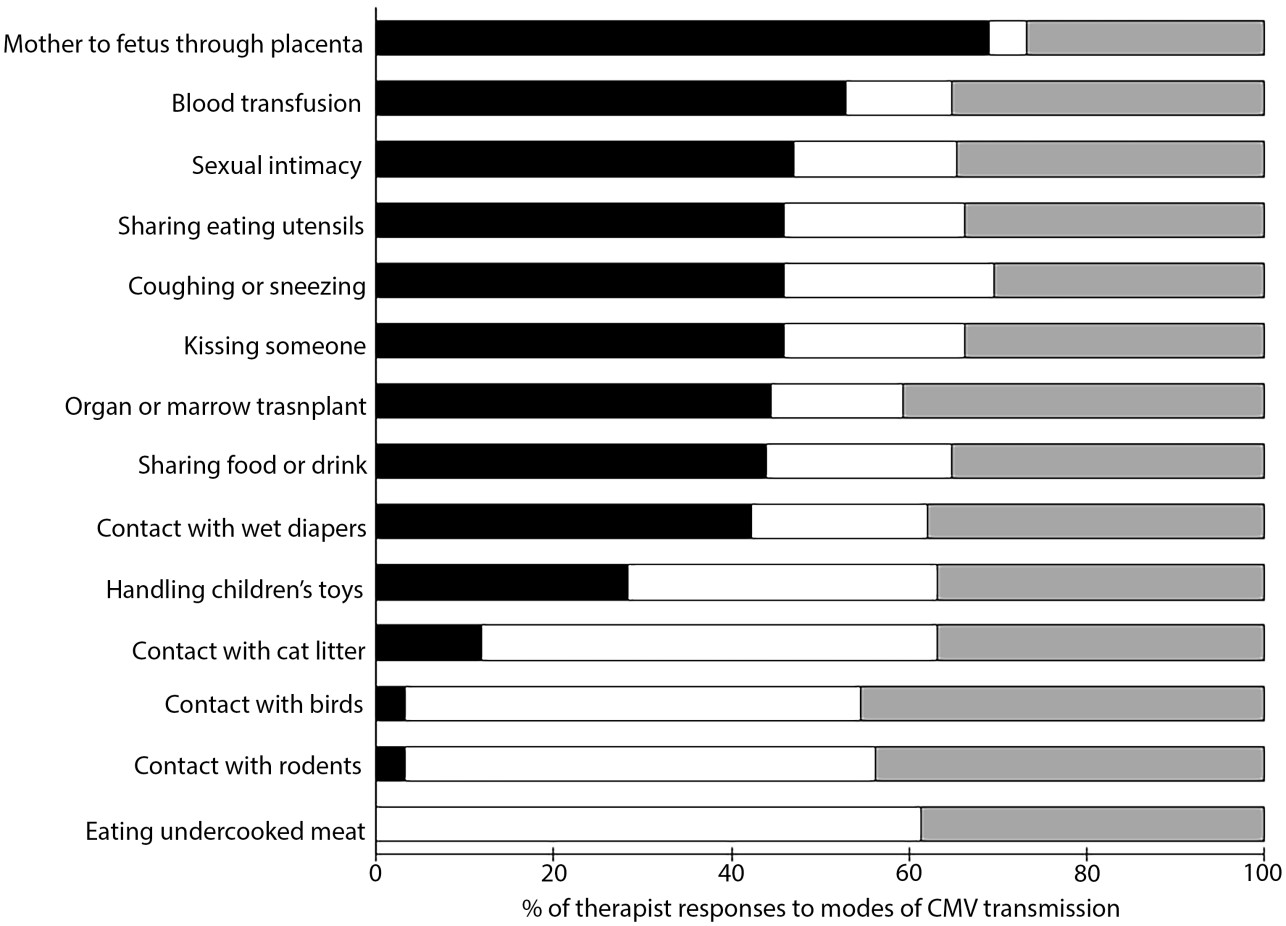
Awareness of modes of transmission of congenital cytomegalovirus (cCMV) amongst physical and occupational therapists. The bars represent the percentage of respondents who answered: yes (black), no (white), or don’t know (grey) to the question, “By which of the following behaviors can people contract or spread cytomegalovirus (CMV) infection?”.

Of 187 respondents, most (69%) indicated that a mother could transmit CMV to the fetus across the placenta (Fig 4). More than half (53%) of respondents correctly indicated that CMV could be transmitted through blood transfusions, followed by correct identification of transmission by organ or marrow transplants (44%). However, 12% incorrectly indicated that CMV could be transmitted by contact with cat litter. The majority of respondents failed to demonstrate understanding of the behavioral modes of CMV transmission. Only 33 respondents (18%) were able to identify all behavioral modes of CMV transmission (Table 2). We considered these respondents to have high HRK of cCMV. The percentage of therapists with high HRK was significantly lower in our sample than the percentage of therapists with high SRF amongst all respondents (18% vs 52%, p < 0.001). Therapists had significantly lower HRK of cCMV than previously published levels of cCMV awareness amongst women who completed college, graduate school, and those with experience as health care workers. The percentage of therapists who had high HRK was significantly greater than awareness levels of women in the general US population (Table 2). When analyzed separately, the percentages of PTs (19% vs 56%, p < 0.001) and OTs (11% vs 39%, p < 0.001) with high HRK was significantly lower than the percentage of respondents who had high SRF of cCMV, respectively.

**Fig 5:**
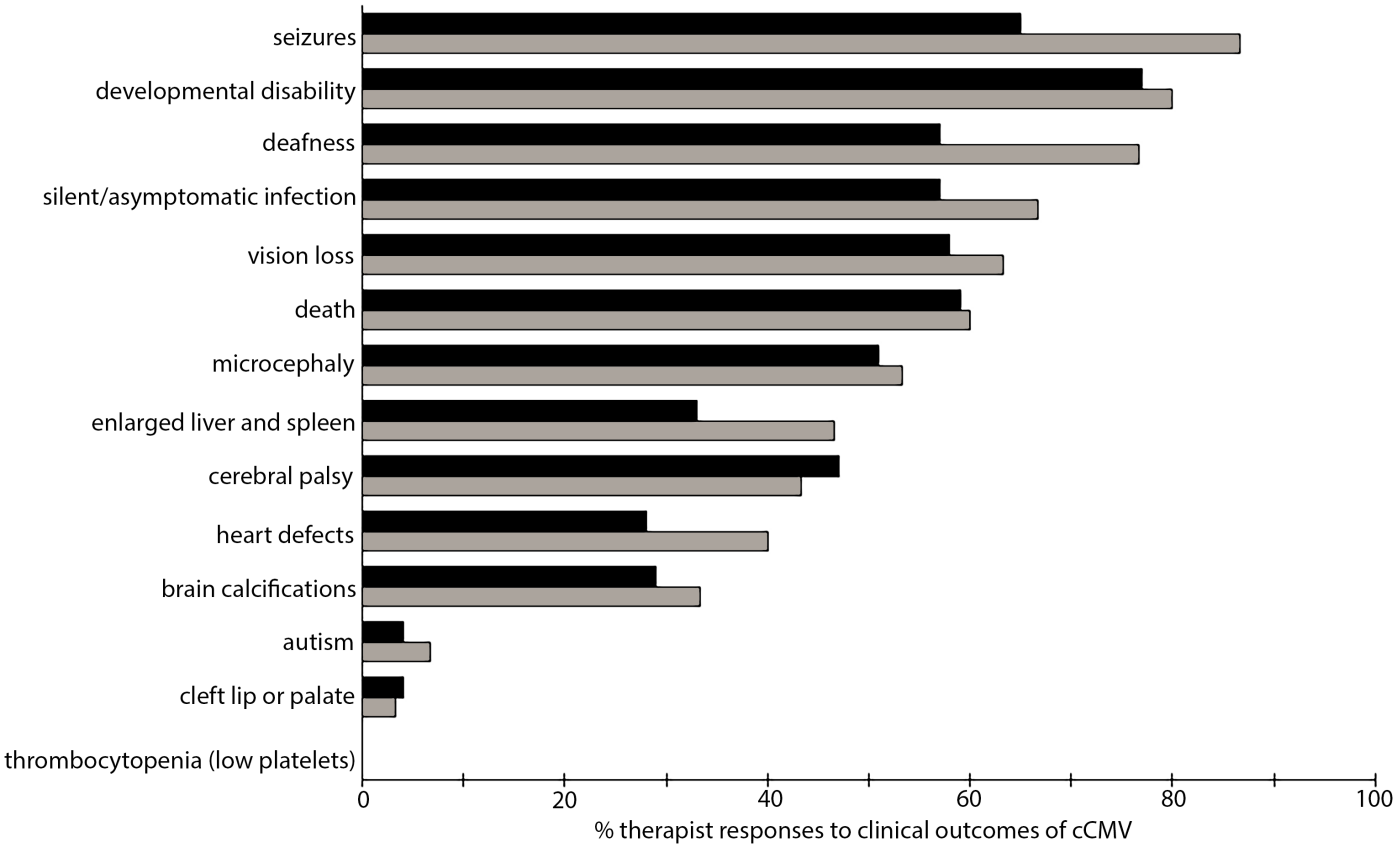
Awareness or knowledge of clinical outcomes of congenital cytomegalovirus (cCMV) amongst physical and occupational therapists. The bars represent percentage of respondents with high SRF (black) and high HRK (grey) who identified each condition as a clinical outcome of cCMV infection. Not all listed conditions are correctly associated with cCMV. No respondents identified thrombocytopenia (low platelets) as a clinical outcome of cCMV. Note that multiple answers were accepted, so the percentages add to more than 100%.

Of respondents who had either high SRF or high HRK, there was a low level of accurate knowledge about clinical manifestations of cCMV. The majority of these respondents could not correctly identify clinical outcomes associated with cCMV disease in the newborn (Fig 5). The median response to questions regarding clinical manifestations of cCMV was “Don’t know”. Most (80% of respondents with high SRF, and 77% of respondents with high HRK) or nearly half (43% of respondents with high SRF, and 47% of respondents with high HRK) correctly associated developmental delay and cerebral palsy as clinical consequences of cCMV, respectively. Few respondents (7% of respondents with high SRF, and 4% of respondents with high HRK) identified autism as a clinical outcome of cCMV. In addition, 28% of respondents with high SRF, and 40% of respondents with high HRK indicated that cCMV could cause congenital heart defects, a symptom not associated with cCMV.

**Fig 6:**
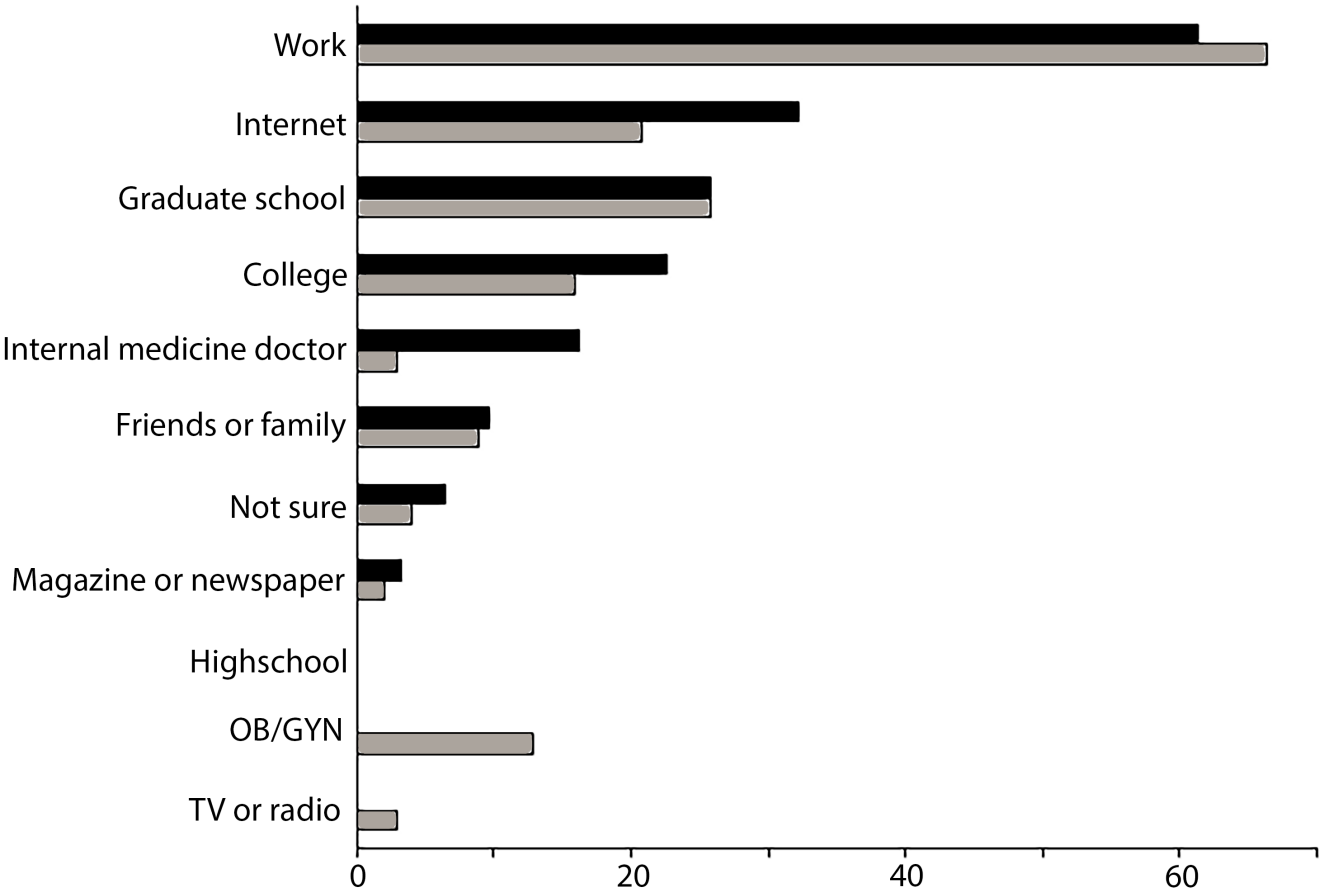
Source of congenital cytomegalovirus (cCMV) awareness or knowledge amongst physical and occupational therapists. The bars represent percentage of respondents with high SRF (black) and high HRK (grey) indicating each source of information. No respondents identified high school as a source of cCMV education. Note that multiple answer were accepted, so the percentages add to more than 100%.

Most participants reported that the source of their information about cCMV was the workplace (66% of respondents with high SRF, and 61% of respondents with HRK, Fig 6). Seven respondents who indicated they received CMV education through the workplace wrote in “other” responses, such as learning about CMV while treating a pediatric patient born with CMV (including a respondent who “treated a child who was shedding the virus, and had to stop treatment while pregnant” and another who indicated that “it had been years since [their practice] have had a patient with CMV”). The next most common source of cCMV information was the internet (21% of respondents with high SRF, and 32% of respondents with high HRK, respectively), followed by graduate school (26% in both groups). Few respondents reported learning about CMV from a doctor (OB/GYN, 13% of respondents with high SRF and 10% of respondents with high HRK; Internal Medicine, 3% of respondents with high SRF only). Only 9% of respondents with high SRF and 16% of respondent with high HRK reported learning about CMV from friends and relatives.

Univariate analyses indicated that working with children, and having a caseload > 75% with children were significantly associated with high HRK of cCMV (Table 3). Although the overall model was significant (OR = 0.214; p < 0.05), individual variables were not significant predictors of cCMV knowledge in the multiple logistic regression analysis. This is likely due to a high degree of collinearity between these variables (χ^2^ [5, 215] = 172.80, p < 0.001). When PTs and OTs were analyzed separately, no demographic variables were significantly associated with HRK of cCMV. This result is also likely due to collinearity in the data, as our sample of PTs were much more likely to work frequently with children than the OT respondents to our survey.

**Table 3:** Self-Reported Knowledge (SRK) and Health Risk Knowledge (HRK) of respondents, grouped by demographic variable (N = 230). Proportion of respondents given in parentheses. Note that not all questions had responses from all participants.

	N	Awareness of cCMV (SRF) N (%)	Demonstrated Understanding of cCMV (HRK) N (%)	OR^a^	95% CI^b^	*P*-value
**Occupation**^c^ **(N = 215)**						
**ALL PTs (reference)**	176 (81.9)	91 (55.5)	28 (18.9)			
**ALL OTs**	39 (18.2)	10 (38.5)	3 (11.1)	1.471	0.41–5.31	0.556
**Gender**^d^ **(N = 184)**						
**Male**	9 (4.9)	2 (22.2)	2 (22.2)	-	-	-
**Female**	170 (93.4)	91 (53.5)	30 (17.6)	-	-	-
**Do not want to specify**	5 (2.7)	1 (33.3)	1 (33.3)	-	-	-
**Age (N = 181)**						
**18–34 (reference)**	44 (24.3)	10 (22.7)	5 (13.2)			
**35–44**	36 (19.9)	21 (58.3)	7 (19.4)	1.883	0.54–6.53	0.319
**45–54**	47 (26.0)	23 (48.9)	12 (25.5)	2.674	0.86–8.35	0.090
**55–64**	47 (26.0)	34 (73.4)	7 (15.4)	1.365	0.40–4.67	0.620
**>65**	7 (3.9)	5 (81.9)	2 (28.8)	3.120	0.473–20.56	0.237
**Ethnicity**^d^ **(N = 184)**						
**Asian**	1 (0.6)	1 (100.0)	0	-	-	-
**Black/African-American**	1 (0.6)	0	0	-	-	-
**Hispanic/Latino**	5 (2.8)	2 (40.0)	1 (20.0)	-	-	-
**Native American/Alaskan**	0 (0)	0	0	-	-	-
**Native Hawaiian/Pacific Islander**	1 (0.6)	1 (100.0)	0	-	-	-
**White**	168 (92.8)	89 (53.0)	31 (18.5)	-	-	-
**Do not want to specify**	10 (5.4)	3 (42.9)	1 (14.3)	-	-	-
**Domestic Status (N = 181)**						
**Single (reference)**	35 (19.3)	14 (40)	4 (11.4)			
**Not single**	146 (80.7)	80 (54.8)	29 (19.9)	1.843	0.60–5.65	0.284
**Parents? (N = 184)**						
**No (reference)**	72 (39.1)	23 (32.9)	10 (14.3)			
**Yes**	112 (60.9)	71 (63.4)	23 (20.4)	1.551	0.69–3.49	0.289
**# of Kids (N = 184)**						
**0 (reference)**	72 (39.1)	35 (50.0)	10 (14.3)			
**1**	17 (9.2)	7 (41.2)	2 (11.8)	0.800	0.16–4.04	0.787
**2**	56 (30.4)	38 (67.9)	12 (21.4)	1.636	0.65–4.13	0.297
**3**	26 (14.1)	16 (61.5)	5 (19.2)	1.429	0.44–4.66	0.555
**4 or more**	13 (7.1)	10 (76.9)	4 (30.8)	2.667	0.69–10.34	0.156
**Work Experience (N = 215)**						
**< 5 years (reference)**	36 (16.7)	8 (25.0)	6 (18.8)			
**5–14.9 years**	44 (20.5)	13 (31.2)	4 (9.8)	0.913	0.34–2.48	0.858
**> 15 years**	135 (62.8)	85 (74.6)	23 (20.2)	0.428	0.14–1.32	0.140
**Work Experience with Kids (N = 210)**						
**0 (reference)**	31 (14.8)	4 (16.7)	1 (4.2)			
**0–4.9 years**	41 (19.5)	11 (31.4)	6 (17.1)	4.759	0.53–42.38	0.162
**5–14.9 years**	58 (27.6)	25 (49.0)	9 (17.6)	4.929	0.59–41.38	0.142
**>15 years**	80 (34.8)	66 (89.1)	17 (23.0)	6.860	0.86–54.58	0.069
**Work with Kids? (N = 215)**						
**No (reference)**	50 (23.3)	9 (21.4)	2 (4.8)			
**Yes**	165 (76.7)	97 (67.0)	31 (21.4)	5.439	1.25–23.76	0.024
**% Caseload with Kids (N = 215)**						
**0% (reference)**	43 (20.0)	0	0			
**1–9.9%**	24 (11.2)	7 (33.3)	4 (19.0)	7.765	0.80–75.01	0.077
**10–24.9%**	4 (1.9)	2 (66.7)	0	0	-	0.999
**25–49.9%**	7 (3.3)	2 (28.6)	1 (14.3)	5.500	0.30–100.47	0.250
**50–74.9%**	7 (3.3)	4 (71.4)	0	0	-	0.999
**75–100%**	130 (60.5)	82 (70.7)	27 (23.3)	0.030	1.31–76.65	0.027
**Contact with Kids (N = 184)**						
**< 1 day/week (reference)**	52 (28.3)	13 (26.0)	5 (10.0)			
**1–2 days/week**	22 (12.1)	14 (63.6)	3 (13.6)	4.421	0.31–6.56	0.652
**3–4 days/week**	27 (14.8)	14 (51.9)	9 (33.3)	4.500	1.33–15.28	0.016
**> 5 days/week**	83 (45.6)	53 (63.9)	16 (19.3)	2.149	0.74–6.28	0.162

^a^OR, odds ratio;

^b^CI, confidence interval

^c^Subfields for PTs and OTs were lumped, due to small sample size in some categories

^d^Univariate analyses were not run for gender or ethnicity, as subcategory response sample sizes were too low for comparisons

## Discussion

Our study is the first to quantify cCMV knowledge in pediatric therapists—a population that is at increased risk of acquiring CMV, is a potential source of spread of the virus to families, and that commonly works with children, including those who have long-term disabilities due to a cCMV infection. Our study is also the first to parcel cCMV knowledge into the distinct subcategories of cCMV awareness (e.g., SRF) and a demonstrated understanding (e.g., HRK) of CMV transmission. Our results support the hypotheses that PTs and OTs have relatively low overall cCMV awareness, but higher awareness than the general population, and equivalent awareness to health care professionals. Only half of the therapists surveyed reported that they were familiar cCMV. It is important to note that even this relatively low level of self-reported awareness does not translate into a demonstrated understanding of CMV transmission. Although most respondents indicated that CMV could be transmitted across the placenta, many fewer therapists were knowledgeable about how to prevent infection—including modes of behavioral transmission that are of relevance to therapeutic practice with young children, such as handling children’s toys. HRK was significantly lower in every comparison than SRF, and was estimated to be only 21% among all respondents. Our results support our hypothesis that higher levels of HRK is associated with greater contact with children, but there was no correlation between HRK and age, specialty, or time in practice. The gap between our respondents’ perception of their cCMV awareness and their demonstrated understanding of CMV transmission is cause for immediate concern.

**Fig 7:**
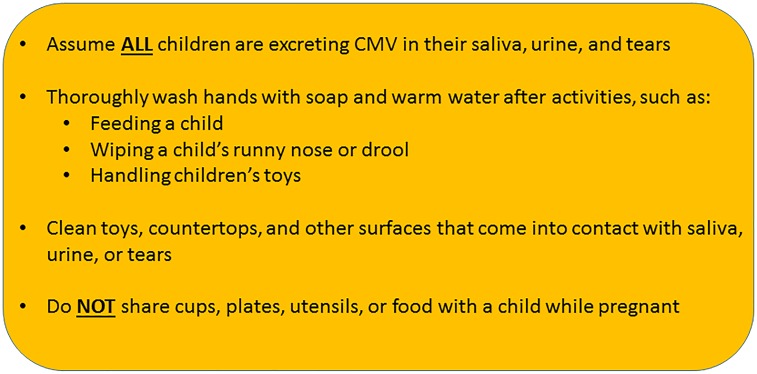
Summary of main behavioral precautions to reduce the risk of cytomegalovirus (CMV) transmission in therapeutic settings.

Congenital CMV is a serious disease, and a leading cause of permanent disability in thousands of children globally each year. Despite the absence of a vaccine, it has been demonstrated that behavioral interventions among pregnant women or women planning a pregnancy, such as improved handwashing and cleansing of surfaces in contact with children’s bodily fluids, could lead to fewer children being affected [40, 41]. Behavioral interventions are particularly important for pediatric therapists, who have intimate contact with children and surfaces that may encounter their saliva or other bodily fluids. Our results validate that therapists need improved cCMV education. Although CMV acquisition rates have not been quantified in pediatric therapists, occupations with similarly intimate levels of interaction with young children, such as daycare educators, kindergarten teachers, and pediatric nurse aides have been demonstrated to be at particularly high risk of having a primary CMV infection during pregnancy [28–36]. Guidelines for good hygienic practices to reduce the risk of CMV infection among PTs and OTs include the following: (1) when interacting with young children, therapists should assume ALL children are secreting CMV in their saliva, urine, and tears; (2) thoroughly wash hands with soap and warm water after activities such as feeding a child, wiping child’s runny nose or drool, handling children’s toys, (3) do not share cups, plates, utensils, toothbrushes or food, (4) clean toys, countertops, and other surfaces that come into contact with saliva, urine, or tears (Fig 7). This list of hygienic procedures is not considered exhaustive (see [71] for full guidelines). It is not possible to avoid all sources of congenital CMV infection, but being aware that contact with all young children can raise the risk of infection may reduce the incidence of cCMV disease. Training programs and disease monitoring interventions aimed at improving hand hygiene behaviors in health care personnel have proven effective at decreasing rates of respiratory and diarrhea infections, as well as HIV [55]. Likewise, studies have shown significant reductions in rates of CMV seroconversion during pregnancy when women are counseled on how to apply correct preventative measures [40, 41].

Of respondents who had either high SRF or high HRK of cCMV, the most commonly reported source of information was the workplace, as has been reported for other populations [72]. Pediatric work environments, such as clinics, schools, or early intervention agencies, could be a key source for disseminating cCMV prevention messages. As evinced by the respondent who reported cessation of treatment to children born with cCMV, communication about the health risks and prevention of CMV transmission needs to be improved. Individuals who oversee clinics and early intervention services should take steps to ensure that therapists are receiving accurate and reliable information about cCMV.

Similar to previous studies, the second most common source of information about cCMV is the internet [61]. The web is a primary source for health information among adults, particularly women [73]. Our results suggest that graduate school curricula are another potentially important source of cCMV information for therapists. The Accreditation Council for Occupational Therapy Education (ACOTE) requires infection control training for accredited doctoral, master’s, and COTA training programs [74]. Similar training does not appear to be required by the Commission on Accreditation in Physical Therapy Education (CAPTE) for PTs, although it is for PTAs [75]. This could mean that although individual PT programs may include infection control, there are no clear standards by which to guide student instruction. In addition, programs are not required to teach students about cCMV, specifically. A study of CMV information contained in books and pregnancy websites, demonstrated that these sources contain accurate, but not adequate CMV information [76]. CMV prevention was included in such references only 50% the time. This proportion is likely even lower in commonly referenced therapy textbooks.

Presenting accurate information to therapists about complications resulting from cCMV is also critical. In our study, the group of respondents with high HRK had a higher percentage of correct responses regarding clinical outcomes caused by cCMV infection than previously demonstrated among women [42, 44]. However, even among people who have factual knowledge about CMV transmission, knowledge about the impact of cCMV disease is low. Among those who had either high SRF or HRK, only a minority could correctly identify outcomes associated with cCMV syndrome. Most respondents associated developmental delays with congenital infection, but many could not identify other symptoms of cCMV and/or incorrectly identified congenital heart defects as caused by cCMV infection. Fetal exposure to CMV greatly increases the risk of a child developing cerebral palsy (CP) [77]; between 5% and 18% of cerebral palsy cases can be attributed to CMV infection [78–80]. Moreover, because cCMV is likely under-diagnosed in the United States [12–17], it is probable that the prevalence of children presenting for therapeutic services with CP due to cCMV is also under-estimated. Yet, CP following symptomatic cCMV infection is often a severe condition involving spasticity in all four limbs, often with associated epilepsy, visual, and/or hearing impairments [79–81]. As such, children with CP secondary to a cCMV infection stand to benefit most by early intervention through physical and occupational therapies [82, 83]. Increased strategic communication about the complications of cCMV infection is warranted.

Interventions for the prevention of cCMV should include structural or environmental approaches [42, 84]. Some studies have suggested that CMV-negative women who are pregnant or wish to become pregnant, and who work in pediatric healthcare or educational settings, should be reassigned to children who were not born with cCMV, who are not shedding the virus, or be removed from working with children altogether [32, 34, 36]. However, avoidance of exposure is difficult, as CMV is ubiquitous in pediatric environments and in the community [85]. Most individuals shedding CMV are asymptomatic, and up to 80% of otherwise healthy children excrete CMV in their bodily fluids [20]. In other words, children who are excreting CMV are indistinguishable from those who are not. Therefore, reassignment into perceived lower risk areas is unlikely to be an effective protection measure [26, 85–87], and may create a false sense of security from an elevated risk of transmission. Pediatric therapists should be advised to assume that children under the age of three have CMV in their urine, saliva, and tears, and practice hygienic precautions [71, 85].

### Limitations

Although online survey research is now routine, data quality may be affected by commonly observed problems associated with use of the internet. We adopted the best research practices developed by [60] when designing our survey to minimize the limitations associated with online research. However, cross talk, in which participants who have completed the survey discuss it with those who have not yet completed the task, could have influenced the naiveté of the participants to the study topic and survey questions. It was impossible to control for this limitation because participants completed the survey in an unsupervised environment. Chandler and Shapiro (2016) have demonstrated that cross talk is a relatively rare occurrence, suggesting its effect on our results are minimal [60].

Our survey was voluntary, and data was derived from a self-selected sample that may not represent all therapists. Our sample size was relatively low, especially among OTs. However, demographic information suggests that our sample was representative of the greater PT population. We did not retest the questionnaire to eliminate ambiguous answers, and some respondents did not follow directions, or guessed at responses. For example, some respondents replied to questions about modes of CMV transmission, despite already answering that they had no familiarity with cCMV. In addition to the inherent bias of self-reported data [50], our survey may overestimate therapist SRF with cCMV. We countered this effect by creating HRK, a variable based only on demonstrated understanding of CMV modes of transmission. Finally, previously published data on cCMV knowledge amongst women with work experience in health care did not specify which fields of practice were encompassed by this group [44]. It is likely this category represents a broad sample of women (e.g., varying educational backgrounds, years of experience, etc). Because this group was inherently different from our sample, our ability to draw direct comparisons was limited.

## Conclusions

Our study demonstrated that there is overall low cCMV knowledge amongst PTs and OTs. The gap between therapist awareness of cCMV their demonstrated understanding of CMV transmission is significant and worrisome. It is important to address this knowledge gap through educational initiatives because cCMV likely represents an occupational hazard for pediatric therapists (e.g., [56]). Furthermore, the success of strategies to prevent cCMV infection includes the active involvement of allied health practitioners involved in mother and child care [88]. Raising cCMV awareness and knowledge among physical and occupational therapists, who may then educate families with whom they are interacting, is an important step in preventing congenital CMV. Such awareness can lead to improvement in preventative behaviors, immediately affecting the burden of cCMV disease.

## Supporting information

S1 Appendix Summary data from survey of health risk knowledge among physical and occupational therapists. (XLSX)

S2 Appendix Survey attrition data for questions 8 and 13 of our results. (XLSX)

## References

[1] Stagno S, Remington J, Klein J. Cytomegalovirus In: Remington JS, Klein JO, editors. Infectious diseases of the fetus and newborn infant. Philadelphia: WB Saunders; 2001 p. 389–424.

[2] Hamprecht K, Maschmann J, Vochem M, Dietz K, Speer CP, Jahn G. Epidemiology of transmission of cytomegalovirus from mother to preterm infant by freastfeeding. Lancet. 2001;357: 513–8. 10.1016/S0140-6736(00)04043-5 11229670

[3] Schleiss MR. Role of breast milk in acquisition of cytomegalovirus infection: Recent Advances. Curr Opin Pediatr. 2006;18: 48–52. 16470162 10.1097/01.mop.0000192520.48411.fa

[4] Boppana SB, Ross SA, Fowler KB. Congenital cytomegalovirus infection: clinical outcome. Clin Infect Dis. 2013;57(suppl 4): S178–S81. 24257422 10.1093/cid/cit629 PMC4471438

[5] Cannon MJ, Schmid DS, Hyde TB. Review of cytomegalovirus seropervalence and demographic characteristics associated with infection. Rev Med Virol. 2010;20: 202–13. 10.1002/rmv.655. 20564615

[6] Centers for Disease Control and Prevention [internet]. Congenital CMV infection trends and statistics 2013. http://www.cdc.gov/cmv/trends-stats.html

[7] Cannon MJ. Congenital cytomegalovirus (CMV) epidemiology and awareness. J Clin Virol. 2009;46S: S6–S10. 10.1016/j.jcv.2009.09.002 19800841

[8] Mestas E. Congenital Cytomegalovirus. Adv Neonatal Care. 2016;16(1): 60–5. 10.1097/ANC.0000000000000242. 26752783

[9] Jones CA. Congenital cytomegalovirus infection. Curr Probl Pediatr Adolesc Health Care. 2003;33(3): 70–93. 10.1067/mps.2003.3. 12605193

[10] De Kegel A, Maes L, Dhooge I, van Hoecke H, De Leenheer E, Van Waelvelde H. Early motor development of children with a congenital cytomegalovirus infection. Res Dev Disabil. 2016;48: 253–61. 10.1016/j.ridd.2015.11.014 26630616

[11] Dollard SC, Grosse SD, Ross DS. New estimates of the prevalence of neurological and sensory sequelae and mortality associated with congenital cytomegalovirus infection. Rev Med Virol. 2007;17(5): 355–63. 10.1002/rmv.544 17542052

[12] Gantt S, Dionne F, Kozak FK, Goshen O, Goldfarb DM, Park AH, et al Cost-effectiveness of universal and targeted newborn screening for congenital cytomegalovirus infection. JAMA Pediatr. 2016;170(12): 1173–80. 10.1001/jamapediatrics.2016.2016 27723885

[13] Fowler KB, McCollister FP, Sabo DL, Shoup AG, Owen KE, Woodruff JL, et al A targeted approach for congenital cytomegalovirus screening within newborn hearing screening. Pediatrics. 2017: e20162128 10.1542/peds.2016-2128 28049114 PMC5260148

[14] Ghekiere S, Allegaert K, Cossey V, Van Ranst M, Cassiman C, Casteels I. Ophthalmological findings in congenital cytomegalovirus infection: when to screen, when to treat? J Pediatr Ophthalmol Strabismus. 2012;49(5): 274–82. 10.3928/01913913-20120710-03 22800795

[15] Duval M, Park AH. Congenital cytomegalovirus: what the otolaryngologist should know. Curr Opin Otolaryngol Head Neck Surg. 2014;22(6): 495–500. 10.1097/MOO.0000000000000104 25222916

[16] Harrison GJ. Current controversies in diagnosis, management, and prevention of congenital cytomegalovirus: updates for the pediatric practitioner. Pediatr Ann. 2015;44(5): e115–e25. 10.3928/00904481-20150512-11 25996198

[17] Çelikel E, Tezer H, Kanik-Yuksek S, Gülhan B, Ozkaya-Parlakay A, Yaralı N. Evaluation of 98 immunocompetent children with cytomegalovirus infection: importance of neurodevelopmental follow-up. Eur J Pediatr. 2015;174(8): 1101–7. 10.1007/s00431-015-2513-9 25762027

[18] Murph JR, Bale JF. The natural-history of acquired cytomegalovirus infection among children in group day care. Am J Dis Child. 1988;142: 843–6. 2839977 10.1001/archpedi.1988.02150080049020

[19] Rosenthal LS, Fowler KB, Boppana SB, Britt WJ, Pass RF, Schmid DS, et al Cytomegalovirus shedding and delayed sensorineural hearing loss: results from longitudinal follow-up of children with congenital infection. Pediatr Infect Dis J. 2009;28(6): 515 10.1097/INF.0b013e318198c724 19483517 PMC2757789

[20] Cannon MJ, Stowell JD, Clark R, Dollard PR, Johnson D, Mask K, et al Repeated measures study of weekly and daily cytomegalovirus shedding patterns in saliva and urine of healthy cytomegalovirus-seropositive children. BMC Infect Dis. 2014;14(1): 569. 25391640 10.1186/s12879-014-0569-1 PMC4240830

[21] Stowell JD, Forlin-Passoni D, Din E, Radford K, Brown D, White A, et al Cytomegalovirus Survival on Common Environmental Surfaces: Opportunities for Viral Transmission. J Infect Dis. 2012;205: 211–4. 10.1093/infdis/jir722 22116837 PMC3276241

[22] Cannon MJ, Hyde TB, Schmid DS. Review of cytomegalovirus shedding in bodily fluids and relevance to congenital cytomegalovirus infection. Rev Med Virol. 2011;21(4): 240–55. 10.1002/rmv.695. 21674676 PMC4494736

[23] Boppana SB, Fowler KB, Britt WJ, Stagno S, Pass RF. Symptomatic congenital cytomegalovirus infection in infants born to mothers with preexisting immunity to cytomegalovirus. Pediatrics. 1999;104(1): 55–60. 10390260 10.1542/peds.104.1.55

[24] Townsend CL, Forsgren M, Ahlfors K, Ivarsson S-A, Tookey PA, Peckham CS. Long-term outcomes of congenital cytomegalovirus infection in Sweden and the United Kingdom. Clin Infect Dis. 2013;56: 1232–9. 10.1093/cid/cit018 23334811 PMC3616516

[25] Bate SL, Dollard SC, Cannon MJ. Cytomegalovirus seroprevalence in the United States: the national health and nutrition examination surveys, 1988–2004. Clin Infect Dis. 2010;50(11): 1439–47. 10.1086/652438 20426575 PMC11000537

[26] Dworsky ME, Welch K, Cassady G, Stagno S. Occupational risk for primary cytomegalovirus infection among pediatric health-care workers. N Engl J Med. 1983;309(16): 950–3. 10.1056/NEJM198310203091604 6312312

[27] Balfour CL, Balfour HHJ. Cytomegalovirus is not an occupational risk for nurses in renal transplant and neonatal uints. Results of a prospective sureillance study. JAMA. 1986;256(14): 1909–14. 3020265

[28] Adler SP. Cytomegalovirus and child day care. Evidence for an increased infection rate among day-care workers. N Engl J Med. 1989;321(19): 1290–6. 10.1056/NEJM198911093211903 2552316

[29] Pass RF, Hutto C, Lyon MD, Cloud G. Increased rate of cytomegalovirus infection among day care center workers. Pediatr Infect Dis J. 1990;9(7): 465–70. 1973533 10.1097/00006454-199007000-00003

[30] Murph JR, Baron JC, Brown CK, Ebelhack CL, Bale JFJ. The occupational risk of cytomegalovirus infection among day-care providers. JAMA. 1991;5: 603–8. 1846215

[31] Bale JFJ, Zimmerman B, Dawson JD, Souza IE, Petheram SJ, Murph JR. Cytomegalovirus transmission in child care homes. Arch Pediatr Adolesc Med. 1999;153(1): 75–9. 9895003 10.1001/archpedi.153.1.75

[32] Kiss P, De Bacquer D, Sergooris L, De Meester M, Vanhoorne M. Cytomegalovirus infection: an occupational hazard to kindergarten teachers working with children aged 2.5–6 years. Int J Occup Environ Health. 2002;8(2): 79–86. 12019684 10.1179/107735202800338966

[33] LePage N, Leroyer A, Cherot-Kornobis N, Lartigau I, Miczek S, Sobaszek A. Cytomegalovirus seroprevalence in exposed and unexposed populations of hospital employees. Eur J Clin Microbiol Infect Dis. 2011;30(1): 65–70. 10.1007/s10096-010-1054-4 20842401

[34] Sobaszek A, Fantoni-Quinton S, Frimat P, Leroyer A, Laynat A, Edme J-L. Prevalence of cytomegalovirus infection among health care workers in pediatric and immunosuppressed adult units. J Occup Environ Med. 2000;42(11): 1109–14. 11094790 10.1097/00043764-200011000-00015

[35] Ford-Jones EL, Kitai I, Davis L, Corey M, Farrell H, Petric M, et al Cytomegalovirus infections in Toronto child-care centers: a prospective study of viral excretion in children and seroconversion among day-care providers. Pediatr Infect Dis J. 1996;15(6): 507–14. 8783347 10.1097/00006454-199606000-00007

[36] Joseph SA, Béliveau C, Muecke CJ, Rahme E, Soto JC, Flowerdew G, et al Cytomegalovirus as an occupational risk in daycare educators. Paediatr Child Health. 2006;11(7): 401 19030309 10.1093/pch/11.7.401 PMC2528629

[37] Marshall BC, Adler SP. The frequency of pregnancy and exposure to cytomegalovirus infections among women with a young child in day care. Am J Obstet Gynecol. 2009;200(2): 163.e1–.e5. 18845286 10.1016/j.ajog.2008.08.037 PMC2662485

[38] Adler SP, Nigro G. Prevention of maternal-fetal transmission of cytomegalovirus. Clin Infect Dis. 2013;57(Supp. 4): S189–S92. 10.1093/cid/cit585 24257425

[39] Stowell JD, Forlin-Passoni D, Radford K, Bate SL, Dollard SC, Bialek SR, et al Cytomegalovirus survival and transferability and the effectiveness of common hand-washing agents against cytomegalovirus on live human hands. Appl Environ Microbiol. 2014;80(2): 455–61. 10.1128/AEM.03262-13 24185855 PMC3911075

[40] Vauloup-Fellous C, Picone O., Cordier AG., Parent-du-Châtelet I, Senat M. V., Frydman R., Grangeot-Keros L. Does hygiene counseling have an impact on the rate of CMV primary infection during pregnancy?: Results of a 3-year prospective study in a French hospital. J Clin Virol. 2009;46: S49–S53. 10.1016/j.jcv.2009.09.003 19811947

[41] Revello MG, Tibaldi C, Masuelli G, Frisina V, Sacchi A, Furione M, et al Prevention of primary cytomegalovirus infection in pregnancy. EBioMedicine. 2015;2: 1205–10. 10.1016/j.ebiom.2015.08.003 26501119 PMC4588434

[42] Ross DS, Victor M, Sumartojo E, Cannon MJ. Women's knowledge of congenital cytomegalovirus: results from the 2005 HealthStyles™ survey. J Womens Health. 2008;17(5): 849–58. 10.1089/jwh.2007.0523 18537486

[43] Pereboom MT, Manniën J, Spelten ER, Schellevis FG, Hutton EK. Observational study to assess pregnant women’s knowledge and behaviour to prevent toxoplasmosis, listeriosis and cytomegalovirus. BMC Pregnancy Childbirth. 2013;13(1): 98. 23627427 10.1186/1471-2393-13-98 PMC3644250

[44] Jeon J, Victor M, Adler SP, Arwady A, Demmler G, Fowler K, et al Knowledge and awareness of congenital cytomegalovirus among women. Infect Dis Obstet Gynecol. 2006;2006: 1–7 10.1155/IDOG/2006/80383 PMC1779612 17485810

[45] Doutré SM, Barrett TS, Greenlee J, White KR. Losing Ground: Awareness of Congenital Cytomegalovirus in the United States. J Early Hear Detect Interv. 2016;1(2): 39–48.

[46] Binda S, Pellegrinelli L, Terraneo M, Caserini A, Primache V, Bubba L, et al What people know about congenital CMV: an analysis of a large heterogeneous population through a web-based survey. BMC Infect Dis. 2016;16(1): 513 10.1186/s12879-016-1861-z 27671033 PMC5037595

[47] Baer HR, McBride HE, Caviness AC, Demmler-Harrison GJ. Survey of congenital cytomegalovirus (cCMV) knowledge among medical students. J Clin Virol. 2014;60(3): 222–42. 10.1037/0003-066X.59.1.29. 10.1037/0003-066X.59.1.29 24794398

[48] Centers for Disease Control and Prevention. Knowledge and practices of obstetricians and gynecologists regarding cytomegalovirus infection during pregnancy—United States, 2007. Morb Mortal Wkly Rep. 2008;57(3):65–8. 18219267

[49] Cordier AG, Guitton S, Vauloup-Fellous C, Grangeot-Keros L, Benachi A, Picone O. Awareness and knowledge of congenital cytomegalovirus infection among health care providers in France. J Clin Virol. 2012;55(2): 158–63. http://dx.doi.org/101016/j.jcv2012.06.022. 22819537 10.1016/j.jcv.2012.06.022

[50] Orne MT. On the social psychology of the psychological experiment: With particular reference to demand characteristics and their implications. Am Psychol. 1962;17(11): 776.

[51] Swanson EC, Schleiss MR. Congenital cytomegalovirus infection: new prospects for prevention and therapy. Pediatr Clin North Am. 2013;60(2): 335–49. 10.1016/j.pcl.2012.12.008 23481104 PMC3807860

[52] American Physical Therapy Association. Physical Therapist Member Demographic Profile 2013 2013. research-dept@apta.org.

[53] American Occupational Therapy Association. Salary and Workforce Survey Executive Summary. The American Occupzational Theraphy Association, Inc; 2015.

[54] Myrhaug HT, Østensjø S, Larun L, Odgaard-Jensen J, Jahnsen R. Intensive training of motor function and functional skills among young children with cerebral palsy: a systematic review and meta-analysis. BMC Pediatr. 2014;14(1): 292. 25475608 10.1186/s12887-014-0292-5 PMC4265534

[55] Marcil WM. Handwashing practices among occupational therapy personnel. Am J Occup Ther. 1993;47(6): 523–8. 8506933 10.5014/ajot.47.6.523

[56] World Confederation for Physical Therapy. Policy Statement: Occupational health and safety of physical therapist. World Confederation for Physical Therapy. 2011: 5.

[57] Flacker J. Occupational therapists' knowledge of cytomegalovirus (cmv) and their use of infection control [Master's thesis]. Puget Sound (OR): University of Puget Sound; 1995.

[58] Cordier A, Guitton S, Vauloup-Fellous C, Grangeot-Keros L, Ayoubi J, Benachi A, et al Awareness of cytomegalovirus infection among pregnant women in France. J Clin Virol. 2012;53(4): 332–7. 10.1016/j.jcv.2011.12.031 22265828

[59] Survey Monkey Inc. San Mateo, CA. www.surveymonkey.com.

[60] Chandler J, Shapiro D. Conducting clinical research using crowdsourced convenience samples. Annu Rev Clin Psychol. 2016;12 10.1146/annurev-clinpsy-021815-093623 26772208

[61] Majima Y, Nishiyama K, Nishihara A, Hata R. Conducting online behavioral research using crowdsourcing services in Japan. Front Psychol. 2017;8 10.3389/fpsyg.2017.00378. PMC5361660 28382006

[62] Chandler J, Mueller P, Paolacci G. Nonnaïveté among Amazon Mechanical Turk workers: Consequences and solutions for behavioral researchers. 2014 10.3758/s13428-013-0365-7 23835650

[63] Crump MJ, McDonnell JV, Gureckis TM. Evaluating Amazon's Mechanical Turk as a tool for experimental behavioral research. PLoS One. 2013;8(3): e57410 10.1371/journal.pone.0057410 23516406 PMC3596391

[64] Downes-Le Guin T, Mechling J, Baker R, editors. Great results from ambiguous sources: Cleaning Internet panel data. ESOMAR World Research Conference: Panel Research; 2006; Amsterdam: ESOMAR.

[65] Buhrmester M, Kwang T, Gosling SD. Amazon's Mechanical Turk: A new source of inexpensive, yet high-quality, data? Perspect Psychol Sci. 2011;6(1): 3–5. 10.1177/1745691610393980 26162106

[66] Mason W, Watts DJ. Financial incentives and the performance of crowds. ACM SigKDD Explorations Newsletter. 2010;11(2):100–8.

[67] Horton JJ, Rand DG, Zeckhauser RJ. The online laboratory: Conducting experiments in a real labor market. Exp Econ. 2011;14(3): 399–425.

[68] IBM Corp. SPSS statistics for windows, version 22.0 Armonk, NY; Released 2013.

[69] Berinsky AJ, Huber GA, Lenz GS. Evaluating online labor markets for experimental research: Amazon. com's Mechanical Turk. Polit Anal. 2012;20(3): 351–68.

[70] Shapiro DN, Chandler J, Mueller PA. Using Mechanical Turk to study clinical populations. Clin Psychol Sci. 2013;1(2): 213–20.

[71] Cannon MJ, Davis KF. Washing our hands of the congenital cytomegalovirus disease epidemic. BMC Public Health. 2005;5(1): 70. 15967030 10.1186/1471-2458-5-70 PMC1182379

[72] Thackeray R, Magnusson BM. Child care provider awareness and prevention of cytomegalovirus and other infectious diseases. Child & Youth Care Forum;45:301–14.

[73] Atkinson NL, Spaerstein SL, Pleis J. Using the internet for health-related activities: Findings from a national probablity sample. J Med Internet Res. 2009;11(1): e4 10.2196/jmir.1035 19275980 PMC2762768

[74] Accreditation Council for Occupational Therapy. Accreditation manual. Bethesda, Maryland; 2017. 163 p.

[75] Commission on Accreditation in Physical Therapy Education. Standards and required elements for accreditation of physical therapist education program. Alexandria, VA; 2016. 34 p.

[76] Thackeray R, Wright A, Chipman K. Congenital cytomegalovirus reference material: A content analysis of coverage and accuracy. Matern Child Health J. 2014;18(3): 584–91. 10.1007/s10995-013-1275-0 23620274 PMC3951885

[77] Gibson CS, MacLennan AH, Goldwater PN, Haan EA, Priest K, Dekker GA. Neurotropic viruses and cerebral palsy: population based case-control study. Br Med J. 2006;332(7533): 76–80. 10.1136/bmj.38668.616806.3A. 16399770 PMC1326927

[78] Alberman E, Peckham C. Cerebral palsy and perinatal exposure to neurotropic viruses: A new study raises further questions about the role of infection. Br Med J. 2006;332(7533): 63 10.1136/bmj.332.7533.63. 16410558 PMC1326917

[79] Smithers-Sheedy H, Raynes-Greenow C, Badawi N, Khandaker G, Menzies R, Jones CA. Cytomegalovirus-related childhood mortality in Australia 1999–2001. J Paediatr Child Health. 2015;51(9): 901 10.1111/jpc.12896 25872417

[80] Smithers-Sheedy H, Raynes-Greenow C, Badawi N, McIntyre S, Jones CA. Congenital cytomegalovirus is associated with severe forms of cerebral palsy and female sex in retrospective population-based study. Dev Med Child Neurol. 2014;56: 846–52. 10.1111/dmcn.12467 24749557

[81] Dakovic I, da Graça Andrada M, Folha T, Neubauer D, Hollody K, Honold M, et al Clinical features of cerebral palsy in children with symptomatic congenital cytomegalovirus infection. Eur J Paediatr Neurol. 2014;18(5): 618–23. 10.1016/j.ejpn.2014.04.007 24931914

[82] Ziviani J, Feeney R, Rodger S, Watter P. Systematic review of early intervention programmes for children from birth to nine years who have a physical disability. Aust Occup Ther J. 2010;57: 210–23. 10.1111/j.1440-1630.2010.00850.x. 20854595

[83] Novak I, McIntyre S, Morgan C, Campbell L, Dark L, Morton N, et al A systematic review of interventions for children with cerebral palsy: state of the evidence. Dev Med Child Neurol. 2013;55: 885–910. 10.111/dmcn.12246. 23962350

[84] Sumartojo E. Structural factors in HIV prevention: Concepts, examples and implications for research. AIDS 2000. 2000;14(Suppl 1): S3. 10.1097/00002030-200006001-00002 10981469

[85] American Academy of Pediatrics. Cytomegalovirus infection. In: Kimberlin DW, Brady MT, Jackson MA, Long SS, editors. Red book: 2015 report of the commitee on infectious diseases. 30th ed. Elk Grove Village: 2015. 6 p.

[86] Chin T, MacGowan A, Jacobson S, Donati M. Viral infections in pregnancy: advice for healthcare workers. J Hosp Infect. 2014;87(1):11–24. 10.1016/j.jhin.2013.12.011 24767811

[87] Lynch L, Spivak ES. The pregnant healthcare worker: fact and fiction. Curr Opin Infect Dis. 2015;28(4): 362–8. 10.1097/QCO.0000000000000180 26098508

[88] Korver AMH, de Vries JJC, de Jong JW, Dekker FW, Vossen ACTM, Oudesluys-Murphy AM. Awareness of congenital cytomegalovirus among doctors in the Netherlands. J Clin Virol. 2009;46S: S11–S5. 10.1016/j.jcv.2009.09.006 19818680

